# Personal

**Published:** 1887-01

**Authors:** 


### PERSONAL.

The editor of this journal desires gratefully to acknowledge the many courtesies extended to him during a late visit to the Central Dental Association of Northern New Jersey. Differences have arisen in the past, but he trusts that in the future they will know him better, as he certainly will them, and that the personal acquaintanceship formed will afford the members half the pleasure it does him.

The society is an active and energetic one, and its influence in elevating the general professional tone in the State has been very marked. Of the intelligence and knowledge usually displayed in the papers and discussions, our readers are competent to judge, for we have before now published much valuable matter from the reports of the society, and are promised much more in the future.

